# Development of Comorbid Depression after Social Fear Conditioning in Mice and Its Effects on Brain Sphingolipid Metabolism

**DOI:** 10.3390/cells12101355

**Published:** 2023-05-10

**Authors:** Iulia Zoicas, Christiane Mühle, Fabian Schumacher, Burkhard Kleuser, Johannes Kornhuber

**Affiliations:** 1 Department of Psychiatry and Psychotherapy, Friedrich-Alexander University Erlangen-Nürnberg (FAU), 91054 Erlangen, Germany; 2 Institute of Pharmacy, Freie Universität Berlin, 14195 Berlin, Germany

**Keywords:** social anxiety, anxiety-like behavior, depressive-like behavior, sphingolipids, acid sphingomyelinase, neutral sphingomyelinase, acid ceramidase, neutral ceramidase, sphingomyelin, ceramide

## Abstract

Currently, there are no animal models for studying both specific social fear and social fear with comorbidities. Here, we investigated whether social fear conditioning (SFC), an animal model with face, predictive and construct validity for social anxiety disorder (SAD), leads to the development of comorbidities at a later stage over the course of the disease and how this affects the brain sphingolipid metabolism. SFC altered both the emotional behavior and the brain sphingolipid metabolism in a time-point-dependent manner. While social fear was not accompanied by changes in non-social anxiety-like and depressive-like behavior for at least two to three weeks, a comorbid depressive-like behavior developed five weeks after SFC. These different pathologies were accompanied by different alterations in the brain sphingolipid metabolism. Specific social fear was accompanied by increased activity of ceramidases in the ventral hippocampus and ventral mesencephalon and by small changes in sphingolipid levels in the dorsal hippocampus. Social fear with comorbid depression, however, altered the activity of sphingomyelinases and ceramidases as well as the sphingolipid levels and sphingolipid ratios in most of the investigated brain regions. This suggests that changes in the brain sphingolipid metabolism might be related to the short- and long-term pathophysiology of SAD.

## 1. Introduction

With a lifetime prevalence of approximately 12% [1], social anxiety disorder (SAD) is the second most common anxiety disorder after specific phobia, and it is characterized by persistent fear and avoidance of social situations [2]. The majority of SAD patients report at least one other psychiatric disorder [3] and about 25% of patients report three or more psychiatric disorders [4]. SAD is highly comorbid with major depressive disorder (between 35 and 70% of SAD patients) and other anxiety disorders, such as specific phobia (14–61%), panic disorder (5–27%), agoraphobia (8–45%) and generalized anxiety disorder (0.6–27%), as well as with alcohol use disorders (up to 50%), obsessive compulsive disorder (2–19%) and posttraumatic stress disorder (3–16%) [3]. Except for specific phobia, SAD has an earlier onset, suggesting that SAD is a risk factor for developing additional psychiatric disorders [3]. The presence of comorbidities is associated with difficulties in diagnosis and treatment and affects the clinical course of SAD. Patients with SAD who have comorbidities show increased symptom severity, treatment resistance, increased risk of relapse and decreased social functioning, and they also have higher rates of suicide when compared with SAD patients without comorbidities [5]. Early diagnosis and intervention might therefore reduce the risk of developing additional psychiatric disorders, while the diagnosis of comorbidities might facilitate an improved treatment outcome. 

Animal models inducing both social anxiety without comorbidities (i.e., specific social anxiety) and social anxiety with comorbidities are needed to investigate the underlying neurobiological mechanism of SAD and its comorbidities. This would provide important information for the development of more specific medication strategies that might lead to an improved treatment outcome. Several paradigms making use of physical stressors, psychological stressors or a combination of both have been shown to induce both social fear and avoidance (as indicative of social anxiety) and additional symptoms in measures of non-social anxiety-like behavior (i.e., generalized anxiety, to be distinguished from social anxiety/social fear) and depressive-like behavior in rodents [6]. Although exposure to these paradigms (e.g., foot shock exposure, restraint stress, chronic mild stress, social instability, social defeat, overcrowding and maternal separation) induces social avoidance and fear, increases non-social anxiety-like behavior and induces a depressive-like phenotype, it is not clear which symptom occurs first. This is relevant information both for the treatment of SAD and for the study of the etiology and pathophysiology of SAD. In order to study SAD without symptoms of generalized anxiety or depressive-like behavior, we developed the social fear conditioning (SFC) paradigm. SFC is an animal model that mimics the major behavioral symptoms of SAD, i.e., reduced social investigation and avoidance of conspecifics as indicative of social fear [7,8], and demonstrates face validity, predictive validity and construct validity for human SAD [7,9]. Although it was shown that SFC induces specific social fear without symptoms of generalized anxiety or depressive-like behavior when assessed 24 h after SFC [7], it is unclear whether such comorbidities might develop at a later stage over the course of the disease.

Several studies indicated that generalized anxiety and depression are related to an altered metabolism of sphingolipids such as sphingomyelin and ceramide [10]. Sphingomyelin is the most abundant sphingolipid in eukaryotes and one of the major components of the plasma membrane. It is composed of phosphorylcholine and ceramide, which in turn consists of a sphingoid backbone with an attached hydrophobic fatty acid chain with a usual chain length of 14 to 26 carbon atoms [11]. Sphingomyelin can be generated by de novo synthesis in a multi-stage process involving multiple enzymes or through the recycling of ceramide by sphingomyelin synthases [12,13]. Ceramide is generated by the hydrolysis of sphingomyelin through the activity of acid sphingomyelinase (ASM), neutral sphingomyelinase (NSM) or alkaline sphingomyelinase with different pH optima [14]. Ceramide can also be generated by de novo synthesis, by degradation of complex (glyco)sphingolipids or through a salvage pathway involving reacylation of the degradation product sphingosine [15,16]. Similar to sphingomyelinases, the ceramidases, i.e., acid ceramidase (AC), neutral ceramidase (NC) and alkaline ceramidase, differ in their pH optimum for the breakdown of ceramide to sphingosine and fatty acid [17]. A delicately balanced sphingolipid metabolism is necessary for neuronal conductivity, viability and synaptic transmission, and many neurodegenerative [18] and psychiatric disorders such as major depressive disorder [19,20,21,22,23,24,25,26,27] are associated with an altered sphingolipid metabolism. 

Clinical studies reported increased ASM activity in peripheral blood mononuclear cells of patients experiencing a major depressive episode [20], and ASM activity was related to depression severity and predicted the improvement of depressive symptoms during therapy [21]. Increased plasma levels of ceramide [22] and several ceramide species, including Cer16:0, Cer18:0, Cer20:0, Cer24:1 and Cer26:1 but not Cer22:0 or Cer24:0, were described in patients experiencing a major depressive episode during the past two years [23]. Increased plasma levels of Cer16:0, Cer18:0, Cer20:0, Cer22:0, Cer24:0 and Cer24:1 were also observed in patients with major depression and bipolar disorder [24], and higher plasma levels of Cer16:0 and Cer18:0 and sphingomyelin SM18:1 were associated with higher severity of depression symptoms in patients with coronary artery disease [25]. In contrast, decreased plasma levels of SM26:1 [26], SM39:1 and SM39:2 [27] but not of SM16:0, SM16:1, SM24:0 and SM24:1 [26] were described in patients with major depressive disorder.

Similar changes in the sphingolipid metabolism were described in animal models of depression. Transgenic mice constitutively overexpressing ASM (ASMtg), for example, showed an increased serum and hippocampal ASM activity and an increased hippocampal ceramide concentration that was associated with increased social and non-social anxiety-like behavior and depressive-like behavior [28,29,30]. Similarly, transgenic mice with restricted overexpression of ASM to the forebrain (ASMtg^fb^) showed increased social anxiety-like behavior and depressive-like behavior [31]. In Wistar rats selectively bred for extremely high anxiety-like behavior (HAB) that show increased non-social anxiety-like and depressive-like behavior similar to ASMtg and ASMtg^fb^ mice but no social anxiety [32], ASM, NSM, AC and NC activities were increased in multiple brain regions associated with anxiety and depression, including the lateral septum, hypothalamus, ventral hippocampus and ventral and dorsal mesencephalon [33]. In the amygdala, however, NC activity was decreased in HAB rats [33]. Exposure to chronic unpredictable stress, an animal model that induces a non-social anxious and depressive-like phenotype [34], increased the levels of Cer16:0, Cer16:1, Cer18:1, Cer22:1 and Cer26:1 but not of Cer18:0, Cer20:0, Cer20:1, Cer22:0, Cer24:0, Cer24:1 and Cer26:0 in the prefrontal cortex in mice [35]. In contrast, the levels of SM16:0, SM20:0, SM22:0, SM24:0 and SM26:0 but not of SM18:0, SM18:1, SM24:1 and SM26:1 were reduced in the prefrontal cortex by chronic unpredictable stress [35], while no sphingolipid changes were observed in the amygdala or cerebellum. Chronic administration of corticosterone, another model known to induce a non-social anxious and depressive-like phenotype [36], also increased Cer22:1 levels in the dorsal hippocampus and Cer20:0, Cer22:1, Cer24:1, Cer26:0 and Cer26:1 levels in the ventral hippocampus. However, the levels of Cer16:0, Cer16:1, Cer18:0, Cer18:1, Cer20:1, Cer22:0, Cer24:0, SM16:0, SM18:0, SM18:1, SM20:0, SM22:0, SM24:0, SM24:1, SM26:0 and SM26:1 in the dorsal and ventral hippocampus were not altered by chronic corticosterone administration [37], suggesting that specific stressors alter the sphingolipid metabolism in a different way. The direct involvement of ceramide in the pathogenesis of anxiety and depression was demonstrated in naïve mice, which developed a depressive-like phenotype after infusion of Cer16 but not Cer8 or Cer20 into the dorsal hippocampus [28,38]. Interestingly, Cer16 induced a non-social anxious phenotype when infused into the basolateral amygdala but not into the dorsal hippocampus, further supporting the fact that ceramides alter depressive-like and anxiety-like behavior in a brain region- and ceramide species-specific way [38].

Together, these studies suggest a fine-tuned balance between the sphingolipid metabolism and affective disorders. However, little is known about the involvement of sphingolipids and sphingolipid metabolizing enzymes in the pathophysiology of SAD with or without comorbid generalized anxiety and/or depression.

In this study, we investigated whether SFC induces social fear and leads to the development of comorbidities at a later stage over the course of the disease and how this affects the brain sphingolipid metabolism. Given that genetic-related strain differences in non-social anxiety-like behavior [39,40], depressive-like behavior [41,42] and social behavior [43,44] were reported, we performed experiments in outbred CD1 mice and in inbred C57BL/6 mice, two mouse lines often used in preclinical research.

## 2. Materials and Methods

### 2.1. Animals

Male CD1 mice and male C57BL/6 mice (Charles River, Sulzfeld, Germany, 10 weeks of age) were individually housed for one week before the experiments started and remained single-housed throughout the experiments. Mice were kept under standard laboratory conditions (12:12 light/dark cycle, lights on at 07:00 h, 22 °C, 60% humidity, food and water ad libitum). Experiments were performed during the light phase between 09:00 and 14:00 in accordance with the Guide for the Care and Use of Laboratory Animals of the Government of Unterfranken (approval code 55.2 DMS-2532-2-314, approval date 13 December 2016) and the guidelines of the NIH. All efforts were made to minimize animal suffering and to reduce the number of animals used. A total number of 36 CD1 mice (*n* = 9 mice/group for the short-term and long-term effects) and a total number of 28 C57BL/6 mice (*n* = 6 mice/group for the short-term effects and *n* = 8 mice/group for the long-term effects) were used for these experiments.

### 2.2. Experimental Overview

**Figure 1:**
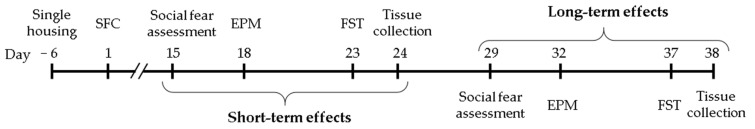
Schematic representation of the experimental protocol. SFC, social fear conditioning; EPM, elevated plus-maze; FST, forced swim test.

After one week of single housing, mice were exposed to SFC to induce social fear (described in Section 2.3). In half of the unconditioned (SFC−) and conditioned (SFC+) mice, the effects of SFC on emotional behavior were assessed starting two weeks after SFC (defined as short-term effects), whereas in the other half of the SFC− and SFC+ mice, testing started four weeks after SFC (defined as long-term effects) (Figure 1). Social fear was assessed in the home cage on day 15 for the short-term effects and on day 29 for the long-term effects. Three days later, the non-social anxiety-like behavior of the mice was tested on the elevated plus-maze (EPM). Five days after EPM, the depressive-like behavior of the mice was tested in the forced swim test (FST). Twenty-four hours later, mice were rapidly killed under CO_2_ anesthesia, and their brains were removed, snap-frozen and stored at −80 °C until further analysis. Twelve brain regions (i.e., the frontal cortex, dorsal and ventral striatum, lateral septum, amygdala, dorsal and ventral hippocampus, thalamus, hypothalamus, dorsal and ventral mesencephalon and cerebellum) were dissected out of coronal brain slices as previously described [31,33]. The activities of ASM and NSM as well as of AC and NC were analyzed from one hemisphere that was counterbalanced between mice. The level of sphingolipids (i.e., sphingomyelin SM16:0, SM18:0, SM20:0, SM22:0, SM24:0 and SM24:1, ceramide Cer16:0, Cer18:0, Cer20:0, Cer22:0, Cer24:0 and Cer24:1 and sphingosine) was quantified from the other hemisphere.

Behavioral experiments were performed in both CD1 and C57BL/6 mice, while the activity of the sphingolipid metabolizing enzymes and the level of sphingolipids were assessed only in CD1 mice given that no SFC-induced behavioral differences were found between these two mouse strains.

### 2.3. Social Fear Conditioning (SFC) Paradigm

To induce social fear, mice were conditioned during SFC using a computerized fear conditioning system (TSE System GmbH, Bad Homburg, Germany) as previously described [7,8,9,45,46,47,48,49,50]; see [6] for a schematic representation of the SFC paradigm. Mice were placed in the conditioning chamber (45 × 22 × 40 cm), and after a 30 s habituation period, an empty wire mesh cage (7 × 7 × 6 cm) was placed as a non-social stimulus near one of the short walls. After 3 min, the non-social stimulus was replaced by an identical cage containing an unfamiliar age- and strain-matched mouse. Unconditioned control mice (SFC−) were allowed to investigate this social stimulus for 3 min, whereas socially fear-conditioned mice (SFC+) were given a 1 s mild electric foot shock (0.7 mA) each time they investigated, i.e., made direct contact with the social stimulus. Mice received between two and four foot shocks with a variable inter-shock interval, depending on when direct social contact was made. The number of foot shocks was assessed as a measure of distress and of social fear learning. Mice were returned to their home cage when no further social contact was made for 2 min (average duration of SFC approximately 10 min). The time that the mice spent investigating the non-social stimulus as a pre-conditioning measure of non-social anxiety was analyzed.

### 2.4. Social Fear Assessment

The SFC-induced social fear was assessed two weeks or four weeks after SFC. Mice were exposed in their home cage to an empty cage identical to the cage used during SFC, to assess non-social investigation as a parameter of non-social fear. After 3 min, the empty cage was replaced by an identical cage containing an unfamiliar age- and strain-matched mouse for an additional 3 min, to assess social investigation as a parameter of social fear. Each stimulus was placed near a short wall of the home cage. The test was recorded and analyzed using JWatcher (V 1.0, Macquarie University, Sydney, Australia and UCLA, Los Angeles, CA, USA). Non-social investigation was defined as direct sniffing of the empty cage, whereas social investigation was defined as direct sniffing of the cage and/or of the social stimulus inside of the cage.

### 2.5. Elevated Plus-Maze (EPM) Test

The non-social anxiety-like behavior of the mice was tested in the EPM as previously described [46,50]. The test was recorded and analyzed using JWatcher. The percentage of time spent on the open arms (150 lx) indicated non-social anxiety-like behavior. The number of entries into the closed arms (30 lx) during the 5 min testing period indicated locomotor activity.

### 2.6. Forced Swim Test (FST)

The depressive-like behavior of the mice was tested in the FST as previously described [7,38]. Mice were individually placed into a Plexiglas cylinder (19 cm diameter, 19 cm height) filled with 25 °C water to a depth of 13 cm for 6 min. The test was recorded and analyzed using JWatcher. An increased percentage of immobility time during the last 4 min of the test indicated a depressive-like phenotype.

### 2.7. Measurement of Sphingomyelinase and Ceramidase Activities

The activity of sphingolipid metabolizing enzymes in the brain tissue was determined using the fluorescent substrate BODIPY-FL-C12-SM (D-7711, Thermo Fisher Scientific, Waltham, MA, USA) for sphingomyelinases and NBD-C12-ceramide (Cay10007958, Cayman, obtained from Biomol, Hamburg, Germany) for ceramidases, with four replicates for each sample based on a previously established method [51] (see Supplementary Materials and Methods).

### 2.8. Sphingolipid Quantification by Liquid Chromatography Tandem Mass Spectrometry (LC-MS/MS)

The brain tissue was subjected to lipid extraction using 1.5 mL methanol/chloroform (2:1, *v:v*) as described before [52,53] (see Supplementary Materials and Methods).

### 2.9. Statistical Analysis

For statistical analysis, SPSS (Version 24, SPSS Inc., Chicago, IL, USA) was used. For each parameter, outliers deviating more than two standard deviations from the mean were excluded from the analysis. Data were analyzed using the Student *t*-test (with reported nominal *p*-values) and two-way ANOVA, followed by Bonferroni’s post hoc analysis. Statistical significance was set at *p* < 0.05. The complete statistical analyses are shown in Tables S1–S4.

## 3. Results

### 3.1. Short-Term Effects of SFC on Social Fear, Non-Social Anxiety-Like Behavior and Depressive-Like Behavior

#### 3.1.1. Outbred CD1 Mice

**Figure 2:**
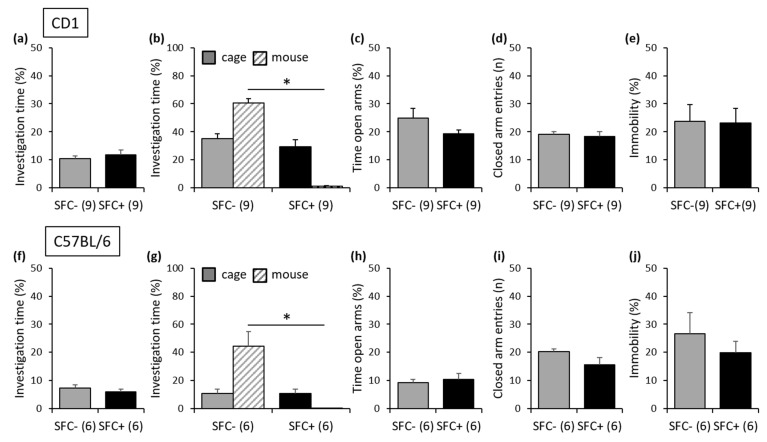
Social fear conditioning (SFC) induces social fear but no short-term alterations in non-social anxiety-like behavior, locomotor activity or depressive-like behavior in outbred CD1 mice and in inbred C57BL/6 mice. (**a**,**f**) Pre-conditioning investigation of the non-social stimulus (empty cage) during SFC on day 1. (**b**,**g**) Investigation of the non-social (empty cage) and social (cage with unknown conspecific) stimuli during social fear assessment on day 15 shown by the unconditioned (SFC−) and conditioned (SFC+) mice. (**c**,**h**) Time spent on the open arms of the elevated plus-maze (EPM) and (**d**,**i**) number of closed-arm entries shown on day 18. (**e**,**j**) Percentage of immobility time in the forced swim test (FST) shown on day 23. Data represent means + SEM, and numbers in parentheses indicate group sizes. * *p* < 0.05.

During SFC, on day 1, SFC+ and SFC− mice spent similar time investigating the non-social stimulus (empty cage), which indicates similar pre-conditioning non-social anxiety between the groups (Figure 2a; Table S1). On day 15, SFC+ and SFC− mice showed similar investigation of the non-social stimulus (empty cage), indicating that SFC did not induce an unspecific non-social fear. However, SFC+ mice showed reduced investigation of the social stimulus (unknown conspecific) compared with SFC− mice, indicating increased social fear (Figure 2b). On day 18, SFC+ and SFC− mice showed similar time spent on the open arms of the EPM (Figure 2c) and a similar number of closed-arm entries (Figure 2d), indicating that SFC did not alter non-social anxiety-like behavior and locomotor activity, respectively. Similarly, on day 23, SFC+ and SFC− mice showed a similar percentage of immobility time in the FST (Figure 2e), indicating that SFC did not induce a depressive-like phenotype after three weeks.

#### 3.1.2. Inbred C57BL/6 Mice

Similar effects to those seen in CD1 mice were observed in C57BL/6 mice, indicating that the genetic-related strain differences did not affect the behavioral phenotype induced by SFC. Therefore, SFC+ and SFC− mice showed similar pre-conditioning non-social anxiety (Figure 2f; Table S1), and SFC induced social fear (day 15; Figure 2g) without altering non-social anxiety-like behavior (day 18; Figure 2h), locomotor activity (day 18; Figure 2i) and depressive-like behavior (day 23; Figure 2j).

### 3.2. Long-Term Effects of SFC on Social Fear, Non-Social Anxiety-like Behavior and Depressive-Like Behavior

#### 3.2.1. Outbred CD1 Mice

**Figure 3:**
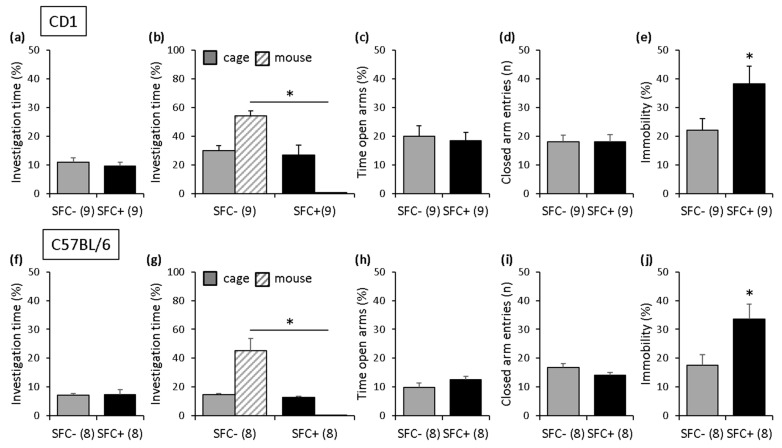
Social fear conditioning (SFC) induces social fear and long-term alterations in depressive-like behavior but not in non-social anxiety-like behavior and locomotor activity in outbred CD1 mice and in inbred C57BL/6 mice. (**a**,**f**) Pre-conditioning investigation of the non-social stimulus (empty cage) during SFC on day 1. (**b**,**g**) Investigation of the non-social (empty cage) and social (cage with unknown conspecific) stimuli during social fear assessment on day 29 shown by the unconditioned (SFC−) and conditioned (SFC+) mice. (**c**,**h**) Time spent on the open arms of the elevated plus-maze (EPM) and (**d**,**i**) number of closed-arm entries shown on day 32. (**e**,**j**) Percentage of immobility time in the forced swim test (FST) shown on day 37. Data represent means + SEM, and numbers in parentheses indicate group sizes. * *p* < 0.05.

During SFC, on day 1, SFC+ and SFC− mice spent similar time investigating the non-social stimulus (empty cage), which indicates similar pre-conditioning non-social anxiety between the groups (Figure 3a; Table S1). On day 29, SFC+ and SFC− mice showed similar investigation of the non-social stimulus (empty cage), indicating that SFC did not induce an unspecific non-social fear. However, SFC+ mice showed reduced investigation of the social stimulus (unknown conspecific) compared with SFC− mice, indicating increased social fear (Figure 3b). On day 32, SFC+ and SFC− mice showed similar non-social anxiety-like behavior (Figure 3c) and similar locomotor activity (Figure 3d) on the EPM. However, on day 37, SFC+ mice showed an increased percentage of immobility time in the FST compared with SFC− mice (Figure 3e), indicating that SFC induced a depressive-like phenotype after five weeks.

Importantly, there was no difference in pre-conditioning non-social anxiety between those CD1 mice tested after two weeks and those CD1 mice tested after four weeks (Table S1) or in the level of social fear shown by the SFC+ mice during social fear assessment. Similarly, there was no difference in the number of foot shocks received by the SFC+ mice during SFC, suggesting that the depressive-like phenotype observed after five weeks is not the result of a stronger conditioning (i.e., better social fear learning) or of a more severe social fear.

#### 3.2.2. Inbred C57BL/6 Mice

Similar effects to those seen in CD1 mice were observed in C57BL/6 mice, indicating that the genetic-related strain differences did not affect the behavioral phenotype induced by SFC. Therefore, SFC+ and SFC− mice showed similar pre-conditioning non-social anxiety (Figure 3f; Table S1), and SFC induced social fear (day 29; Figure 3g) without altering non-social anxiety-like behavior (day 32; Figure 3h) and locomotor activity (day 32; Figure 3i). However, like in the outbred CD1 mice, SFC induced a depressive-like phenotype in inbred C57BL/6 mice (day 37; Figure 3j) after five weeks, which was not the result of a stronger conditioning (i.e., better social fear learning) or of a more severe social fear. Therefore, there was no difference in pre-conditioning non-social anxiety between those C57BL/6 mice tested after two weeks and those C57BL/6 mice tested after four weeks or in the level of social fear shown by the SFC+ mice during social fear assessment, and there was no difference in the number of foot shocks received by the SFC+ mice during SFC (Table S1).

### 3.3. Strain Differences in Behavior

Separate statistics revealed a lower social investigation and non-social investigation during social fear assessment and a higher anxiety-like behavior on the EPM in SFC− C57BL/6 mice compared with SFC− CD1 mice (SFC− mice used to assess the short- and long-term effects were combined), suggesting strain differences in social behavior and in non-social anxiety-like behavior in unconditioned mice (Table S1). However, the locomotor activity and the depressive-like behavior of SFC− C57BL/6 and SFC− CD1 mice were similar.

### 3.4. Short-Term Effects of SFC on Brain Sphingolipid Metabolizing Enzymes

**Figure 4:**
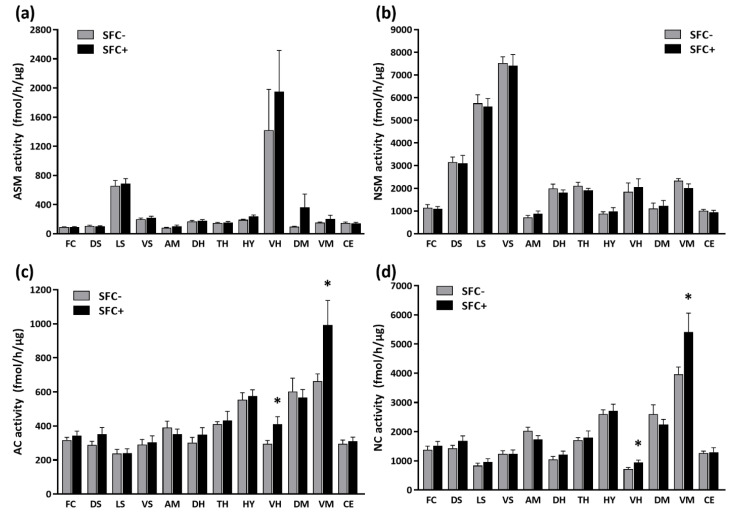
Specific social fear alters the activity of the brain sphingolipid metabolizing enzymes in CD1 mice. The activity of acid and neutral sphingomyelinases (ASM in (**a**), NSM in (**b**)) as well as of acid and neutral ceramidases (AC in (**c**), NC in (**d**)) was analyzed in the frontal cortex (FC), dorsal striatum (DS), lateral septum (LS), ventral striatum (VS), amygdala (AM), dorsal hippocampus (DH), thalamus (TH), hypothalamus (HY), ventral hippocampus (VH), dorsal mesencephalon (DM), ventral mesencephalon (VM) and cerebellum (CE) of unconditioned (SFC−) and conditioned (SFC+) mice (*n* = 9 mice/group). Data represent means + SEM. * *p* < 0.05.

Three weeks after SFC, SFC+ and SFC− mice showed a similar ASM (Figure 4a) and NSM (Figure 4b) activity in all investigated brain regions (Table S2). SFC+ mice showed an increased AC and NC activity in the ventral hippocampus (+39%) and ventral mesencephalon (+49%) compared with SFC− mice (Figure 4c and Figure 4d, respectively), suggesting that specific social fear is accompanied by an increased activity of ceramidases especially in the ventral hippocampus and ventral mesencephalon.

### 3.5. Short-Term Effects of SFC on Brain Sphingolipids

Three weeks after SFC, SFC+ mice showed increased levels of SM24:1 in the dorsal hippocampus (+18%) compared with SFC− mice, whereas no other changes in sphingolipid levels (Table S3) and sphingolipid ratios (i.e., the percentage of a certain SM or Cer species compared to total SM or total Cer, respectively; Table S4) were observed.

### 3.6. Long-Term Effects of SFC on Brain Sphingolipid Metabolizing Enzymes

**Figure 5:**
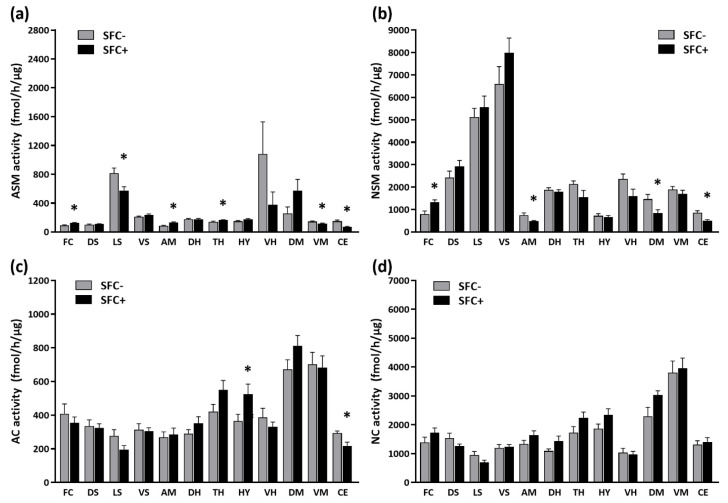
Social fear with comorbid depression alters the activity of the brain sphingolipid metabolizing enzymes in CD1 mice. The activity of acid and neutral sphingomyelinases (ASM in (**a**), NSM in (**b**)) as well as of acid and neutral ceramidases (AC in (**c**), NC in (**d**)) was analyzed in the frontal cortex (FC), dorsal striatum (DS), lateral septum (LS), ventral striatum (VS), amygdala (AM), dorsal hippocampus (DH), thalamus (TH), hypothalamus (HY), ventral hippocampus (VH), dorsal mesencephalon (DM), ventral mesencephalon (VM) and cerebellum (CE) of unconditioned (SFC−) and conditioned (SFC+) mice (*n* = 9 mice/group). Data represent means + SEM. * *p* < 0.05.

Five weeks after SFC, the activity of the sphingolipid metabolizing enzymes was altered in several brain regions (Table S2). SFC+ mice showed an increased ASM activity in the frontal cortex (+36%), amygdala (+60%) and thalamus (+16%), as well as a decreased ASM activity in the lateral septum (−29%), ventral mesencephalon (−19%) and cerebellum (−53%) compared with SFC− mice (Figure 5a). SFC+ mice also showed an increased NSM activity in the frontal cortex (+65%) and a decreased NSM activity in the amygdala (−36%), dorsal mesencephalon (−43%) and cerebellum (−42%) (Figure 5b), as well as an increased AC activity in the hypothalamus (+44%) and a decreased AC activity in the cerebellum (−26%) (Figure 5c). This suggests that social fear with comorbid depression is accompanied especially by an altered activity of sphingomyelinases.

### 3.7. Long-Term Effects of SFC on Brain Sphingolipids

Five weeks after SFC, SFC+ mice showed decreased absolute levels of Cer16:0 (−29%), Cer18:0 (−19%), Cer22:0 (−15%) and total ceramide (−19%) in the frontal cortex, sphingosine in the dorsal hippocampus (−29%), and SM16:0 (−17%), SM18:0 (−15%), Cer18:0 (−28%), Cer20:0 (−19%), Cer22:0 (−15%), Cer24:0 (−20%) and total ceramide (−26%) in the thalamus, as well as increased levels of SM24:1 in the thalamus (+34%), SM18:0 in the dorsal mesencephalon (+24%) and SM20:0 in the ventral mesencephalon (+24%) (Table S3), and changes in sphingolipid ratios (Table S4) compared with SFC− mice.

### 3.8. Effects of Duration of Single Housing on Brain Sphingolipid Metabolizing Enzymes, Sphingolipid Levels and Sphingolipid Ratios

To investigate whether the length of the single housing affects the activity of the sphingolipid metabolizing enzymes, we compared the activity of ASM, NSM, AC and NC in SFC− mice from both experiments (three weeks after SFC versus five weeks after SFC). Longer single housing (45 days versus 31 days) was accompanied by a decreased ASM activity in the hypothalamus (−22%), NSM activity in the ventral mesencephalon (−19%), AC activity in the hypothalamus (−34%) and amygdala (−32%) and NC activity in the hypothalamus (−28%) and amygdala (−34%) (Table S2).

Longer single housing (45 days versus 31 days) was also accompanied by increased absolute levels of SM24:0 in the dorsal hippocampus (+45%) and decreased levels of SM22:0 (−31%), SM24:1 (−30%), Cer24:1 (−19%) and sphingosine (−18%) in the ventral mesencephalon (Table S3) as well as by changes in sphingolipid ratios (Table S4).

## 4. Discussion

This study shows for the first time that SFC alters emotional behavior and the brain sphingolipid metabolism in a time-point-dependent manner. Social fear is induced as an initial symptom and is not accompanied by changes in non-social anxiety-like behavior, locomotor activity and depressive-like behavior for at least two to three weeks after SFC. After five weeks, however, SFC+ mice also show a depressive-like phenotype, suggesting that depression develops as comorbidity at a later stage over the course of the disease. These different pathologies were accompanied by different alterations in the brain sphingolipid metabolism. While specific social fear was accompanied by an increased activity of ceramidases, especially in the ventral hippocampus and ventral mesencephalon, and increased levels of SM24:1 in the dorsal hippocampus, social fear with comorbid depression was accompanied by changes in the activity of sphingomyelinases and acid ceramidase, as well as in sphingolipid levels and sphingolipid ratios in most of the investigated brain regions.

### 4.1. Development of Comorbid Depression after SFC in Male Mice

In previous studies, we have shown that SFC induces specific social fear in male CD1 mice without altering non-social anxiety-like behavior 24 h [7] and three weeks [9] after SFC or depressive-like behavior and locomotor activity 24 h after SFC [7]. We now confirmed and extended these findings by showing that social fear remains specific for at least two to three weeks. After five weeks, however, a depressive-like phenotype developed in SFC+ mice. These findings suggest that the SFC paradigm is an appropriate animal model for investigating both specific social fear and social fear with comorbid depression and might be used for comparative studies without having a biasing effect due to the use of two different paradigms that might differentially alter emotional behavior. Even though between 0.6 and 27% of SAD patients also develop symptoms of generalized anxiety [3], SFC+ mice did not show an increased non-social anxiety-like behavior for at least four weeks after SFC. This might indicate that generalized anxiety does not develop as a comorbidity at this time point or that the number of animals used in this study and in [9] was too low to detect the individuals that might develop generalized anxiety as a comorbidity of SAD. Symptoms of generalized anxiety may however arise later along the course of the disease in SFC+ mice, an aspect that will be investigated in future studies.

Importantly, these effects were observed both in outbred CD1 mice and in inbred C57BL/6 mice and suggest that the genetic-related strain differences do not affect the behavioral phenotype induced by SFC, demonstrating the reliability of the SFC paradigm. Although the SFC-induced effects on emotional behavior were similar between these two strains of mice, basal differences in non-social anxiety-like behavior [39,40], depressive-like behavior [41,42] and social behavior [43,44] were previously reported. For example, naïve C57BL/6 mice are more anxious and show higher levels of depressive-like behavior and a more affiliative type of social behavior. While no differences in non-aggressive social behavior were observed between C57BL/6 and CD1 mice, CD1 mice engaged more in aggressive behaviors, resulting in a higher total time spent in social interactions [43,44]. Although we did not find strain differences in depressive-like behavior in this study, we could replicate the differences in non-social anxiety-like behavior and social behavior.

### 4.2. Effects of SFC on Brain Sphingolipid Metabolizing Enzymes

Regarding the effects of SFC on the brain sphingolipid metabolizing enzymes, we observed increased AC and NC activities in the ventral hippocampus and ventral mesencephalon in mice expressing specific social fear. Besides modulating emotional behavior and cognitive functions, the hippocampus regulates fear-related behaviors together with the prefrontal cortex, lateral septum and amygdala [54,55] and processes contextual information, i.e., information related to the environment of a fearful situation [55]. The ventral mesencephalon also regulates emotional behavior, including aversive responses and social avoidance [56]. It is therefore feasible that the increased AC and NC activities in the ventral hippocampus and ventral mesencephalon observed in SFC+ mice three weeks after SFC relate to social fear. Five weeks after SFC, when SFC+ mice also developed a comorbid depression in addition to social fear, we observed more extensive alterations in the activity of the brain sphingolipid metabolizing enzymes. While the activity of ceramidases was only slightly affected, the activity of sphingomyelinases was altered in multiple brain regions. Although no studies to date investigated the activity of sphingomyelinases and ceramidases in SAD and social fear, several studies described an increased ASM activity in animal models of depression [28,30,31,33]. An increased ASM activity in the prefrontal cortex and amygdala was also described in ASMtg^fb^ mice that show a depressive-like phenotype [31], suggesting that an increased ASM activity within these brain regions might relate to depressive-like behavior. To our surprise, we did not observe an increased ASM activity in the dorsal or ventral hippocampus in SFC+ mice, although this was previously reported in ASMtg^fb^ mice [31]. However, in HAB rats that also show a depressive-like phenotype like ASMtg^fb^ mice, ASM activity was increased in the ventral hippocampus but not in the dorsal hippocampus [33], suggesting a differential contribution of the hippocampal ASM to depressive-like behavior in different animal models of depression. We also found several brain regions where the activity of the sphingolipid metabolizing enzymes was decreased in SFC+ mice with comorbid depression, out of which the cerebellum strikes with reduced activity of both sphingomyelinases and ceramidases, i.e., ASM, NSM and AC. Although classically involved in motor control, the cerebellum also modulates the reward circuitry and social behavior, and several studies demonstrated that impaired cerebellar function is associated with impaired social behavior and depressive-like behavior [57,58]. Given that a delicately balanced sphingolipid metabolism is necessary for neuronal conductivity, viability and synaptic transmission [18], it is feasible that a reduced ASM, NSM and AC activity alters the functionality of the cerebellum and therefore reflects the behavioral deficits observed specifically in mice expressing social fear with comorbid depression.

### 4.3. Effects of SFC on Brain Sphingolipids

Not only the activity of the sphingolipid metabolizing enzymes but also the sphingolipid levels and sphingolipid ratios were altered in mice expressing specific social fear and social fear with comorbid depression. As only the SM24:1 levels were increased in the dorsal hippocampus in mice expressing specific social fear (i.e., three weeks after SFC), the question arises whether the increased activity of ceramidases observed in these mice might take longer to sufficiently affect the sphingolipid levels. Mice expressing social fear and comorbid depression (i.e., five weeks after SFC) showed an altered brain sphingolipid composition, most profoundly at the level of the thalamus and frontal cortex. Given that the thalamus is part of the limbic system that mediates the formation and expression of emotional responses, including social avoidance and mood, by integrating and redistributing emotionally relevant stimuli via reciprocal connections to the frontal cortex, hippocampus and amygdala [59], these sphingolipid changes might underlie the behavioral changes observed in SFC+ mice five weeks after SFC.

In very simplified terms, an increased activity of sphingomyelinases should lead to decreased sphingomyelin and increased ceramide levels, whereas an increased activity of ceramidases should result in decreased ceramide and increased sphingosine levels. Although this was observed in the dorsal mesencephalon, ventral mesencephalon and partly in the thalamus, in other brain regions the sphingolipid changes cannot be solely explained by the altered activity of sphingomyelinases and ceramidases. The sphingolipid changes observed at the level of the frontal cortex and thalamus suggest that the activity of other enzymes involved in the ceramide turnover might be altered in mice expressing social fear and comorbid depression. For example, a decreased mRNA expression of glucosylceramidase 2 (*Gba2*) was observed in ASMtg^fb^ mice that show a depressive-like phenotype [31]. Given that glucosylceramidase 2 converts complex glucosylceramides into ceramide [60], a decreased *Gba2* expression would result in decreased ceramide levels. Further enzymes involved in regulating ceramide levels and known to be affected in neuropsychiatric disorders (e.g., alkaline ceramidase and sphingomyelin synthases [13]) might also play a role and could be altered in specific brain regions, counterbalancing or increasing the effects of the analyzed sphingomyelinases and ceramidases.

### 4.4. Effects of Prolonged Single Housing on Behavior and Brain Sphingolipid Metabolism

We also investigated whether the length of the single housing affects the sphingolipid metabolism in SFC− mice. Although we did not see any changes in behavior caused by the prolonged single housing (45 days versus 31 days), alterations in sphingolipid metabolizing enzymes appeared evident, especially at the level of the hypothalamus and amygdala, brain regions involved in emotional behavior, social behavior and non-social anxiety-like behavior. In social organisms like mice and rats, social interactions are reinforcing, and social isolation exerts strong effects on the brain and behavior [61]. While most studies have shown that single housing exerts anxiogenic effects in female rodents [62], socially housed male rodents often engage in aggressive interactions in their home cage, which may explain why most data suggest that social isolation has anxiolytic effects in males [61,62,63], but see [64]. Given that an increased ASM, AC and NC activity was found in the hypothalamus of HAB rats that show high levels of non-social anxiety, it is possible that the reduced ASM, AC and NC activities observed in SFC− mice after 45 days of single housing reflect changes at the level of the brain that are not yet expressed at the behavioral level as reduced non-social anxiety. In addition, the sphingolipid levels and sphingolipid ratios were altered in the dorsal hippocampus, ventral hippocampus and ventral mesencephalon, suggesting that prolonged single housing alters the sphingolipid composition of the brain before behavioral changes occur.

## 5. Conclusions

In summary, we have shown that in both outbred CD1 mice and inbred C57BL/6 mice, the social fear induced by SFC remains specific for at least two to three weeks while a comorbid depressive-like phenotype develops after five weeks. Whereas specific social fear was accompanied by an increased activity of ceramidases in the ventral hippocampus and ventral mesencephalon and by relatively small changes in sphingolipid levels in the dorsal hippocampus, social fear with comorbid depression was accompanied by changes in the activity of sphingomyelinases and acid ceramidase, as well as in sphingolipid levels and sphingolipid ratios in most of the investigated brain regions. This suggests that changes in sphingolipid metabolism might be related to the short- and long-term pathophysiology of SAD.

## Data Availability

The data generated during the current study are available from the corresponding author on request.

## Supplementary Materials

The following supporting information can be downloaded at: https://www.mdpi.com/article/10.3390/cells12101355/s1, Supplementary Materials and Methods; Table S1: Complete statistics on the behavioral data; Table S2: Complete statistics on the activity of sphingolipid metabolizing enzymes; Table S3: Complete statistics on the absolute sphingolipid levels; Table S4: Complete statistics on the sphingolipid ratios.
