# Supplementary material for: Development of Comorbid Depression after Social Fear Conditioning in Mice and Its Effects on Brain Sphingolipid Metabolism
Source: Cells. 2023 May 10;12(10):1355. doi: 10.3390/cells12101355 (PMC10216690; doi:10.3390/cells12101355)
Supplement: Supplementary file 1 [file cells-12-01355-s001.zip › cells-2296254-supplementary.pdf]

Supplementary Materials and Methods

Measurement of Sphingomyelinase and Ceramidase Activities

The activity of sphingolipid metabolizing enzymes was determined using the fluorescent substrate BODIPY-FL-C12-SM (D-7711, Thermo Fisher Scientific, Waltham, MA, USA) for sphingomyelinases and NBD-C12-ceramide (Cay10007958, Cayman, obtained from Biomol, Hamburg, Germany) for ceramidases, with four replicates for each sample based on a previously established method [51]. The brain tissue was homogenized in lysis reagent (250 mM sucrose, 1 mM EDTA, 0.2 % Triton X-100, 1 x Roche protease inhibitor cocktail; approximately 200 µl/10 mg tissue) using a Tissue Lyser LT bead mill (Qiagen, Hilden, Germany) with steal beads followed by freezing at −80 °C to enhance lysis. Supernatants obtained after thawing, ultrasound treatment (water bath for 60 sec) and centrifugation at 16000 *g* at 4 °C for 10 min were diluted 1:4 in lysis reagent and used for activity assays and for protein determination (Bradford/Coomassie kit, Thermo Fisher Scientific, Waltham, MA, USA). A standard enzyme reaction in a 96 well polystyrene plate contained 58 pmol sphingomyelin or 50 pmol ceramide as a substrate in a total volume of 50 µl of reaction buffer of the following composition: 200 mM sodium acetate buffer (pH 5.0), 500 mM NaCl, 0.2 % IGEPAL® CA-630 (NP 40) detergent for ASM, 200 mM HEPES buffer (pH 7.0), 200 mM $MgCl_2$, 0.05 % IGEPAL® CA-630 (NP 40) for NSM; 200 mM sodium acetate buffer (pH 4.5), 100 mM NaCl, 0.03 % IGEPAL® CA-630 (NP 40) for AC and 200 mM HEPES (pH 7.0), 100 mM NaCl, 0.03 % IGEPAL® CA-630 (NP 40) for NC. The reaction was initiated by the addition of 2 µl of tissue lysate corresponding to 0.5−1 µg protein. After incubation at 37 °C for 1−18 h, depending on enzymatic activity, reactions were stopped by freezing at −20 °C and stored until further processing. To separate product and uncleaved substrate, 1.5 µl of each reaction were directly spotted on silica gel 60 thin layer chromatography plates (ALUGRAM SIL G, 818232, Macherey-Nagel, Düren, Germany) and separated using ethyl acetate with 1 % (v/v) acetic acid as a solvent. Signals were detected on a Typhoon Trio scanner (488 nm excitation, 520 nm emission, 325−385 V, 100 µm resolution, GE Healthcare Life Sciences, Buckinghamshire, UK) and quantified with the ImageQuant software (GE Healthcare Life Sciences, Buckinghamshire, UK). Enzymatic activities were calculated as the hydrolysis rate of sphingomyelin or ceramide (fmol), respectively, per time (h) and per protein (µg).

Sphingolipid Quantification by Liquid Chromatography Tandem Mass Spectrometry (LC-MS/MS)

Brain tissue was subjected to lipid extraction using 1.5 ml methanol/chloroform (2:1, v:v) as described before [52]. The extraction solvent contained d_7-sphingosine (d_7-Sph), C17:0 ceramide (Cer17:0) and d_{31}-C16:0 sphingomyelin (d_{31}-SM16:0) (all Avanti Polar Lipids, Alabaster, USA) as internal standards. Chromatographic separations were achieved on a 1290 Infinity II HPLC (Agilent Technologies, Waldbronn, Germany) equipped with a Poroshell 120 EC-C8 column (3.0 × 150 mm, 2.7 µm; Agilent Technologies). MS/MS analyses were carried out using a 6495 triple-quadrupole mass spectrometer (Agilent Technologies) operating in the positive electrospray ionization mode (ESI+) [53]. The following mass transitions were recorded (qualifier product ions in parentheses): *m*/*z* 300.3 → 282.3 (252.3) for sphingosine, *m*/*z* 307.3 → 289.3 (259.3) for d_7-Sph, *m*/*z* 520.5 → 264.3 (282.3) for Cer16:0, *m*/*z* 534.5 → 264.3 (282.3) for Cer17:0, *m*/*z* 548.5 → 264.3 (282.3) for Cer18:0, *m*/*z* 576.6 → 264.3 (282.3) for Cer20:0, *m*/*z* 604.6 → 264.3 (282.3) for Cer22:0, *m*/*z* 630.6 → 264.3 (282.3) for Cer24:1, *m*/*z* 632.6 → 264.3 (282.3) for Cer24:0, *m*/*z* 703.6 → 184.1 (86.1) for SM16:0, *m*/*z* 731.6 → 184.1 (86.1) for SM18:0, *m*/*z* 734.6→ 184.1 (86.1) for d_{31}-SM16:0, *m*/*z* 759.6 → 184.1 (86.1) for SM20:0, *m*/*z* 787.7 → 184.1 (86.1) for SM22:0, *m*/*z* 813.7 → 184.1 (86.1) for SM24:1, and *m*/*z* 815.7 → 184.1 (86.1) for SM24:0. Peak areas of ceramide and sphingomyelin subspecies, as determined with MassHunter software (version 10.1, Agilent Technologies), were normalized to those of the internal standards (Cer17:0 or d_{31}-SM16:0) followed by external calibration in the range of 1 fmol to 50 pmol on column. Sphingosine was directly quantified via its deuterated internal standard d_7-Sph (0.25 pmol on column). Determined sphingolipid amounts were normalized to the actual protein content (as determined by Bradford assay) of the tissue homogenate used for extraction (pmol/mg protein).

Table S1. Complete statistics on the behavioral data. Pre-conditioning non-social anxiety is reflected by the time spent investigating the non-social stimulus (empty cage) during social fear conditioning (SFC) on day 1. Non-social anxiety-like behavior is reflected by the percentage of time spent on the open arms of the elevated plus maze (EPM). Locomotor activity is reflected by the number of entries into the closed arms of the EPM. Depressive-like behavior is reflected by the percentage of immobility time during the forced swim test (FST). SFC– mice, unconditioned control mice; SFC+ mice, socially fear conditioned mice. Data were analyzed using the Student t-test and two-way ANOVA, followed by a Bonferroni's post-hoc analysis. Statistical significance was set at $p<0.05$.

**Short-term effects of SFC; comparison between SFC– and SFC+ mice starting two weeks after SFC**		
Outbred CD1 mice	**Conditioning effect**	**Conditioning x stimulus effect**
Pre-conditioning non-social anxiety (Figure 2a)	T(16)=-0.62; p=0.545	
Social fear assessment (Figure 2b)	F(1,32)=98.56; **p<0.001***	F(1,32)=66.93; **p<0.001***
Non-social anxiety-like behavior on the EPM (Figure 2c)	T(16)=1.47; p=0.161	
Locomotor activity on the EPM (Figure 2d)	T(16)=0.62; p=0.542	
Depressive-like behavior in the FST (Figure 2e)	T(16)=0.08; p=0.934	
Inbred C57BL/6 mice	**Conditioning effect**	**Conditioning x stimulus effect**
Pre-conditioning non-social anxiety (Figure 2f)	T(10)=0.95; p=0.363	
Social fear assessment (Figure 2g)	F(1,20)=14.74; **p=0.001***	F(1,20)=14.88; **p=0.001***
Non-social anxiety-like behavior on the EPM (Figure 2h)	T(10)=-0.44; p=0.667	
Locomotor activity on the EPM (Figure 2i)	T(10)=1.69; p=0.121	
Depressive-like behavior in the FST (Figure 2j)	T(10)=0.77; p=0.459	
**Long-term effects of SFC; comparison between SFC– and SFC+ mice starting four weeks after SFC**		
Outbred CD1 mice	**Conditioning effect**	**Conditioning x stimulus effect**
Pre-conditioning non-social anxiety (Figure 3a)	T(16)=0.42; p=0.680	
Social fear assessment (Figure 3b)	F(1,30)=35.96; **p<0.001***	F(1,30)=37.39; **p<0.001***
Non-social anxiety-like behavior on the EPM (Figure 3c)	T(16)=0.19; p=0.855	
Locomotor activity on the EPM (Figure 3d)	T(16)=-0.06; p=0.953	
Depressive-like behavior in the FST (Figure 3e)	T(16)=-2.17; **p=0.045***	
Inbred C57BL/6 mice	**Conditioning effect**	**Conditioning x stimulus effect**
Pre-conditioning non-social anxiety (Figure 3f)	T(14)=-0.15; p=0.881	
Social fear assessment (Figure 3g)	F(1,28)=30.33; **p<0.001***	F(1,28)=25.82; **p<0.001***
Non-social anxiety-like behavior on the EPM (Figure 3h)	T(14)=-1.32; p=0.207	
Locomotor activity on the EPM (Figure 3i)	T(14)=1.61; p=0.129	
Depressive-like behavior in the FST (Figure 3j)	T(14)=-2.47; **p=0.027***	
**Comparison between SFC+ mice tested starting two weeks after SFC and SFC+ mice tested starting four weeks after SFC**		
Outbred CD1 mice	**Time-point effect**	**Conditioning x time-point effect**
Pre-conditioning non-social anxiety	F(1,32)=0.67; p=0.419	F(1,32)=0.54; p=0.469
Foot shocks received during SFC	T(16)=-0.54; p=0.597	
Social fear shown by SFC+ mice (social investigation during social fear assessment)	T(15)=1.34; p=0.201	
Inbred C57BL/6 mice	**Time-point effect**	**Conditioning x time-point effect**
Pre-conditioning non-social anxiety	F(1,24)=0.21; p=0.662	F(1,24)=0.49; p=0.491
Foot shocks received during SFC	T(12)=-0.71; p=0.487	
Social fear shown by SFC+ mice (social investigation during social fear assessment)	T(12)=0.06; p=0.953	
**Strain differences in behavior; comparison between SFC– C57BL/6 mice and SFC– CD1 mice**		
Social investigation during social fear assessment	T(30)=2.08; **p=0.047***	
Non-social investigation during social fear assessment	T(30)=5.97; **p<0.001***	
Non-social anxiety-like behavior on the EPM	T(30)=3.92; **p<0.001***	
Locomotor activity on the EPM	T(30)=0.16; p=0.875	
Depressive-like behavior in the FST	T(30)=0.28; p=0.780	

Table S2. Complete statistics on the activity of sphingolipid metabolizing enzymes in the frontal cortex (FC), dorsal striatum (DS), lateral septum (LS), ventral striatum (VS), amygdala (AM), dorsal hippocampus (DH), thalamus (TH), hypothalamus (HY), ventral hippocampus (VH), dorsal mesencephalon (DM), ventral mesencephalon (VM) and cerebellum (CE). ASM, acid sphingomyelinase; NSM, neutral sphingomyelinase; AC, acid ceramidase; NC, neutral ceramidase. Data were analyzed using the Student t-test. Statistical significance was set at $p<0.05$. Red indicates increased activity of sphingolipid metabolizing enzymes whereas blue indicates decreased activity of sphingolipid metabolizing enzymes in socially fear conditioned (SFC+) mice compared with unconditioned control (SFC−) mice, or in longer single housed SFC− mice compared with shorter single housed SFC− mice (45 days versus 31 days). The percentual change in activity is indicated after the p value.

**Short-term effects of SFC; comparison between SFC– and SFC+ mice three weeks after SFC**												
**Enzyme**	**FC**	**DS**	**LS**	**VS**	**AM**	**DH**	**TH**	**HY**	**VH**	**DM**	**VM**	**CE**
ASM	T(16)=0.138; p=0.948	T(16)=0.662; p=0.517	T(16)=-0.304: p=0.765	T(16)=-0.669; p=0.513	T(15)=-0.956; p=0.354	T(15)=-0.594; p=0.561	T(16)=-0.466; p=0.647	T(16)=-1.976; p=0.066	T(15)=-0.665; p=0.516	T(15)=-1.349; p=0.197	T(15)=-1.094; p=0.291	T(16)=0.208; p=0.838
NSM	T(16)=0.261; p=0.798	T(16)=0.141; p=0.889	T(14)=0.272; 0.789	T(16)=-0.350; p=0.731	T(15)=-1.008; p=0.330	T(15)=0.837; p=0.415	T(15)=1.039; p=0.315	T(16)=-0.578; p=0.571	T(16)=-0.428; p=0.674	T(1)=-0.359; p=0.725	T(14)=1.569; p=0.139	T(14)=0.685; p=0.504
AC	T(16)=-0.852; p=0.407	T(16)=-1.463; p=0.163	T(15)=-0.052; p=0.959	T(16)=-0.282; p=0.781	T(16)=0.830; p=0.419	T(14)=-0.942; p=0.362	T(15)=-0.357; p=0.726	T(15)=-0.372: p=0.715	T(15)=-2.530; **p=0.023; +39%**	T(14)=0.397; p=0.697	T(14)=-2.177; **p=0.047; +49%**	T(15)=-0.448; p=0.661
NC	T(16)=-0.762; p=0.457	T(15)=-1.279; p=0.220	T(16)=-0.889; p=0.387	T(16)=-0.020, p=0.985	T(15)=1.586; p=0.133	T(15)=-1.048; p=0.311	T(15)=-0.364; p=0.721	T(16)=-0.422; p=0.679	T(15)=-2.221; **p=0.042; +33%**	T(16)=0.966; p=0.348	T(15)=-2.163; **p=0.047; +37%**	T(14)=-0.176; p=0.863
**Long-term effects of SFC; comparison between SFC– and SFC+ mice five weeks after SFC**												
**Enzyme**	**FC**	**DS**	**LS**	**VS**	**AM**	**DH**	**TH**	**HY**	**VH**	**DM**	**VM**	**CE**
ASM	T(15)=-2.742; **p=0.015; +36%**	T(14)=-0.692; p=0.500	T(15)=2.599; **p=0.020; -29%**	T(16)=-1.402; p=0.180	T(15)=-4.257; **p=0.001; +60%**	T(16)=-0.169; p=0.868	T(15)=-2.480; **p=0.025; +16%**	T(14)=-1.865; p=0.083	T(14)=1.459; p=0.167	T(15)=-1.725; p=0.105	T(16)=2.379; **p=0.030; -19%**	T(15)=3.743; **p=0.002; -53%**
NSM	T(16)=-2.997; **p=0.009; +65%**	T(15)=-1.276; p=0.221	T(15)=-0.676; p=0.509	T(16)=-1.653; p=0.118	T(15)=2.421; **p=0.029; -36%**	T(14)=0.571; p=0.577	T(15)=1.686; p=0.112	T(14)=0.435; p=0.670	T(15)=1.949; p=0.070	T(16)=2.440; **p=0.027; -43%**	T(16)=0.890; p=0.387	T(15)=3.488; **p=0.003; -42%**
AC	T(15)=0.833; p=0.418	T(16)=0.240; p=0.813	T(16)=-1.388; p=0.184	T(16)=0.251; p=0.805	T(15)=-0.331; p=0.745	T(16)=-1.300; p=0.212	T(16)=-1.872; p=0.080	T(15)=-2.138; **p=0.049; +44%**	T(16)=0.892; p=0.386	T(15)=-1.659; p=0.118	T(16)=0.199; p=0.845	T(14)=2.893; **p=0.012; -26%**
NC	T(16)=-1.388; p=0.184	T(15)=1.427; p=0.174	T(15)=1.687; p=0.112	T(16)=-0.251; p=0.805	T(14)=-1.599; p=0.132	T(15)=-1.852; p=0.084	T(16)=-1.741; p=0.101	T(11)=-1.828; p=0.089	T(16)=0.313; p=0.758	T(15)=-2.006; p=0.063	T(16)=-0.272; p=0.789	T(16)=-0.433; p=0.671
**Effects of duration of single housing; comparison between SFC– mice three weeks after SFC and SFC– mice five weeks after SFC**												
**Enzyme**	**FC**	**DS**	**LS**	**VS**	**AM**	**DH**	**TH**	**HY**	**VH**	**DM**	**VM**	**CE**
ASM	T(16)=-0.209; 0.837	T(15)=0.492; p=0.630	T(16)=-1.503; p=0.152	T(16)=-0.744; p=0.468	T(14)=-0.162; p=0.873	T(15)=-0.292; p=0.774	T(16)=0.332; p=0.744	T(15)=3.156; **p=0.007; -22%**	T(14)=0.475; p=0.642	T(14)=-1.800; p=0.093	T(16)=0.379; p=0.710	T(16)=-0.121; p=0.905
NSM	T(16)=1.752; p=0.099	T(16)=2.046; p=0.058	T(14)=1.154; p=0.268	T(16)=1.121; p=0.279	T(15)=-0.245; p=0.810	T(14)=0.576; p=0.574	T(15)=-0.143; p=0.889	T(15)=1.171; p=0.260	T(15)=1.097; p=0.290-	T(16)=-1.107; p=0.285	T(15)=2.586; **p=0.021; -19%**	T(15)=1.435; p=0.172
AC	T(15)=-1.605; p=0.129	T(16)=-1.111; p=0.283	T(16)=-0.879; p=0.392	T(16)=-0.500; p=0.624	T(15)=2.438; **p=0.028; -32%**	T(15)=0.315; p=0.757	T(15)=-0.215; p=0.833	T(15)=3.280; **p=0.005; -34%**	T(16)=-1.548; p=0.141	T(15)=-0.717; p=0.484	T(15)=-0.460; p=0.652	T(14)=0.078; p=0.939
NC	T(16)=-0.041; p=0.968	T(16)=-0.569; p=0.578	T(16)=-0.742; p=0.469	T(16)=0.213; p=0.834	T(15)=3.846; **p=0.002; -34%**	T(15)=-0.346; p=0.734	T(15)=-0.059; p=0.954	T(15)=3.255; **p=0.005; -28%**	T(16)=-1.941; p=0.070	T(16)=0.683; p=0.504	T(16)=0.329; p=0.747	T(15)=-0.270; p=0.791

Table S3. Complete statistics on the absolute sphingolipid levels in the dorsal hippocampus (DH), hypothalamus (HY), ventral hippocampus (VH), ventral mesencephalon (VM), frontal cortex (FC), amygdala (AM), thalamus (TH), dorsal mesencephalon (DM) and cerebellum (CE). Data were analyzed using the Student t-test. Statistical significance was set at p<0.05. Red indicates increased sphingolipid levels whereas blue indicates decreased sphingolipid levels in socially fear conditioned (SFC+) mice compared with unconditioned control (SFC−) mice, or in longer single housed SFC− mice compared with shorter single housed SFC− mice (45 days versus 31 days). The percentual change in sphingolipid levels is indicated after the p value.

Short-term effects of SFC; comparison between SFC– and SFC+ mice three weeks after SFC

Brain region	SM16:0	SM18:0	SM20:0	SM22:0	SM24:0	SM24:1	Total SM	Cer16:0	Cer18:0	Cer20:0	Cer22:0	Cer24:0	Cer24:1	Total Cer	Sphingosine
DH	T(16)=-0.938; p=0.362	T(15)=-0.792; p=0.440	T(16)=-0.609; p=0.551	T(15)=-1.015; p=0.326	T(16)=-1.711; p=0.106	T(14)=-2.273; p=0.039; +18%	T(14)=-1.173; p=0.260	T(15)=-1.930; p=0.073	T(16)=-0.984; p=0.340	T(15)=-0.062; p=0.951	T(16)=-1.629; p=0.123	T(15)=-2.098; p=0.053	T(15)=-2.030; p=0.061	T(15)=-1.333; p=0.202	T(16)=0.696; p=0.497
HY	T(15)=-1.240; p=0.234	T(16)=-0.297; p=0.771	T(16)=-0.144; p=0.887	T(15)=-1.779; p=0.095	T(16)=-1.565; p=0.137	T(16)=-1.283; p=0.218	T(15)=-1.253; p=0.230	T(15)=1.502; p=0.154	T(16)=0.499; p=0.625	T(15)=-0.362; p=0.722	T(15)=-0.998; p=0.334	T(16)=-0.248; p=0.807	T(15)=-1.092; p=0.292	T(15)=0.352; p=0.730	T(16)=-0.275; p=0.787
VH	T(16)=-0.326; p=0.749	T(15)=-0.051; p=0.960	T(16)=-0.171; p=0.866	T(16)=0.421; p=0.679	T(16)=-0.443; p=0.663	T(15)=0.287; p=0.778	T(15)=0.033; p=0.974	T(16)=-0.213; p=0.834	T(15)=-0.662; p=0.518	T(16)=0.200; p=0.844	T(16)=-0.453; p=0.657	T(15)=-1.351; p=0.197	T(14)=-1.158; p=0.266	T(14)=-0.456; p=0.655	T(16)=-0.705; p=0.491
VM	T(14)=1.224; p=0.241	T(15)=0.093; p=0.927	T(15)=-0.699; p=0.495	T(15)=0.175; p=0.863	T(14)=1.140; p=0.273	T(15)=-0.702; p=0.493	T(14)=0.040; p=0.580	T(15)=1.067; p=0.303	T(14)=1.906; p=0.077	T(14)=1.534; p=0.147	T(14)=1.773; p=0.098	T(14)=1.902; p=0.078	T(14)=-0.969; p=0.349	T(14)=0.719; p=0.974	T(15)=-0.131; p=0.103

Long-term effects of SFC; comparison between SFC– and SFC+ mice five weeks after SFC

Brain region	SM16:0	SM18:0	SM20:0	SM22:0	SM24:0	SM24:1	Total SM	Cer16:0	Cer18:0	Cer20:0	Cer22:0	Cer24:0	Cer24:1	Total Cer	Sphingosine
FC	T(14)=-0.693; p=0.500	T(15)=-0-012; p=0.990	T(15)=0.799; p=0.437	T(14)=1.411; p=0.180	T(15)=0.208; p=0.838	T(16)=0.524; p=0.607	T(14)=-0.702; p=0.494	T(15)=3.821; p=0.002; -29%	T(15)=3.052; p=0.008; -19%	T(16)=1.484; p=0.157	T(16)=2.410; p=0.028, -15%	T(16)=1.910; p=0.074	T(16)=1.699; p=0.109	T(15)=3.108; p=0.007; -19%	T(16)=-0.912; p=0.375
AM	T(15)=-0.956; p=0.354	T(15)=-0.581; p=0.570	T(14)=0.486; p=0.634	T(15)=-1.805; p=0.091	T(15)=-0.514; p=0.597	T(14)=-1.034; p=0.319	T(14)=-0.843; p=0.414	T(15)=0.260; p=0.798	T(15)=0.662; p=0.518	T(15)=1.045; p=0.313	T(15)=0.356; p=0.727	T(14)=-0272; p=0.789	T(15)=-1.178; p=0.257	T(15)=0.531; p=0.603	T(15)=-1.460; p=0.165
DH	T(16)=0.817; p=0.426	T(15)=0.648; p=0.527	T(15)=1.320; p=0.207	T(16)=0.652; p=0.524	T(16)=1.778; p=0.094	T(15)=1.293; p=0.216	T(15)=0.996; p=0.335	T(16)=1.448; p=0.167	T(16)=1.568; p=0.132	T(16)=1.501; p=0.153	T(15)=2.036; p=0.060	T(16)=1.661; p=0.116	T(15)=1.947; p=0.071	T(15)=1.875; p=0.080	T(15)=2.642; p=0.018; -29%
TH	T(14)=4.054; p=0.001: -17%	T(16)=2.180; p=0.045; -15%	T(15)=-0.530; p=0.604	T(16)=-0.815; p=.0427	T(16)=-0.575; p=0.574	T(16)=-2.840; p=0.012; +34%	T(14)=1.519; p=0.151	T(16)=2.034; p=0.059	T(16)=2.583; p=0.020; -28%	T(16)=2.199; p=0.043; -19%	T(15)=2.343; p=0.033; -15%	T(15)=3.387; p=0.004; -20%	T(14)=-0.255; p=0.803	T(14)=2.474; p=0.027; -26%	T(15)=-0.739; p=0.471
HY	T(16)=-1.334; p=0.201	T(15)=-0.904; p=0.380	T(16)=-0.694; p=0.498	T(15)=-0.549; p=0.591	T(15)=0.410; p=0.688	T(16)=0.923; p=0.370	T(15)=-0.939; p=0.363	T(16)=1.608; p=0.127	T(15)=1.194; p=0.251	T(16)=0.343; p=0.736	T(16)=0.021; p=0.984	T(16)=0.738; p=0.471	T(16)=0.647; p=0.527	T(15)=1.106; p=0.286	T(16)=1.929; p=0.072
VH	T(16)=0.538; p=0.598	T(15)=0.542; p=0.596	T(15)=1.352; p=0.196	T(16)=0.285; p=0.779	T(15)=0.460; p=0.652	T(16)=1.346; p=0.197	T(15)=0.963; p=0.351	T(16)=-0.018; p=0.986	T(16)=0.709; p=0.489	T(16)=0.682; p=0.505	T(16)=0.284; p=0.780	T(15)=-0.643; p=0.530	T(15)=1.283; p=0.219	T(15)=0.604; p=0.555	T(16)=1.101; p=0.287
DM	T(16)=-1.205; p=0.246	T(16)=-3.007; p=0.008: +24%	T(16)=-0.138; p=0.892	T(14)=0.817; p=0.428	T(16)=-0.258; p=0.800	T(14)=1.390; p=0.186	T(14)=-2.022; p=0.063	T(15)=-0.585; p=0.567	T(16)=-0.789; p=0.442	T(16)=-1.891; p=0.077	T(16)=-2.031; p=0.059	T(15)=-1.709; p=0.108	T(16)=-0.455; p=0.655	T(15)=-0.366; p=0.720	T(15)=0.020; p=0.984
VM	T(16)=0.067; p=0.948	T(15)=-0.573; p=0.575	T(14)=-3.022; p=0.009; +24%	T(15)=-2.044; p=0.059	T(15)=-1.889; p=0.078	T(15)=-1.565; p=0.138	T(14)=-1.847; p=0.086	T(15)=1.133; p=0.275	T(15)=0.623; p=0.543	T(15)=1.665; p=0.117	T(16)=0.127; p=0.900	T(16)=-0.930; p=0.366	T(16)=-1.174; p=0.258	T(15)=0.251; p=0.805	T(16)=-0.878; p=0.393
CE	T(9)=-0.354; p=0.732	T(8)=-0.884; p=0.402	T(8)=-1.612; p=0.146	T(9)=0.027; p=0.979	T(9)=0.182; p=0.859	T(9)=0.362; p=0.726	T(8)=-0.778; p=0.459	T(9)=0.326; p=0.752	T(9)=-0.287; p=0.781	T(9)=-0.557; p=0.591	T(9)=-0.614; p=0.554	T(8)=-1.286; p=0.234	T(9)=0.024; p=0.981	T(8)=-0.799; p=0.447	T(8)=-0.954; p=0.368

Effects of duration of single housing; comparison between SFC– mice three weeks after SFC and SFC– mice five weeks after SFC

Brain region	SM16:0	SM18:0	SM20:0	SM22:0	SM24:0	SM24:1	Total SM	Cer16:0	Cer18:0	Cer20:0	Cer22:0	Cer24:0	Cer24:1	Total Cer	Sphingosine
DH	T(16)=-1.249; p=0.230	T(16)=0.513; p=0.615	T(16)=-0.782; p=0.446	T(15)=-1.491; p=0.157	T(16)=-2.531; p=0.022; +45%	T(15)=-1.692; p=0.111	T(15)=-0.303; p=0.766	T(15)=-0.566; p=0.580	T(16)=1.281; p=0.219	T(16)=0.354; p=0.728	T(16)=-0.709; p=0.489	T(16)=-0.273; p=0.788	T(15)=-1.104; p=0.287	T(15)=0.816; p=0.427	T(16)=-0.428; p=0.674
HY	T(15)=-0.336; p=0.742	T(16)=-0.720; p=0.482	T(16)=0.318; p=0.755	T(14)=1.495; p=0.157	T(15)=1.353; p=0.196	T(16)=0.698; p=0.495	T(15)=-0.850; p=0.409	T(16)=-0.596; p=0.560	T(16)=-0.470; p=0.644	T(15)=-1.174; p=0.259	T(15)=-1.137; p=0.273	T(16)=-0.411; p=0.686	T(15)=-0.598; p=0.559	T(15)=-0.540; p=0.597	T(16)=0.309; p=0.761
VH	T(16)=-0.395; p=0.698	T(15)=1.646; p=0.121	T(16)=-0.105; p=0.918	T(16)=-0.032; p=0.975	T(16)=-0.371; p=0.716	T(16)=-1.381; p=0.186	T(15)=0.483; p=0.344	T(16)=-0.124; p=0.903	T(15)=0.088; p=0.931	T(16)=1.221; p=0.240	T(16)=0.881; p=0.391	T(15)=2.009; p=0.063	T(15)=-1.560; p=0.140	T(15)=-0.037; p=0.971	T(16)=-1.035; p=0.316
VM	T(16)=-0.519; p=0.611	T(15)=-0.984; p=0.341	T(15)=-0.403; p=0.692	T(15)=2.358; p=0.032; -31%	T(15)=1.539; p=0.145	T(15)=2.538; p=0.023; -30%	T(15)=0.095; p=0.637	T(16)=1.319; p=0.206	T(16)=1.851; p=0.083	T(16)=-0.944; p=0.359	T(16)=-0.394; p=0.699	T(16)=1.376; p=0.188	T(15)=2.543; p=0.022: -19%	T(16)=1.864; p=0.081	T(16)=2.366; p=0.031; -18%

Table S4. Complete statistics on the sphingolipid ratios (i.e., the percentage of a certain sphingomyelin (SM) or ceramide (Cer) species compared to total SM or total Cer, respectively), sphingomyelin to ceramide ratios and ceramide to sphingosine ratios in the dorsal hippocampus (DH), hypothalamus (HY), ventral hippocampus (VH), ventral mesencephalon (VM), frontal cortex (FC), amygdala (AM), thalamus (TH), dorsal mesencephalon (DM) and cerebellum (CE). Data were analyzed using the Student t-test. Statistical significance was set at p<0.05. Red indicates increased sphingolipid ratios whereas blue indicates decreased sphingolipid ratios in socially fear conditioned (SFC+) mice compared with unconditioned control (SFC−) mice, or in longer single housed SFC− mice compared with shorter single housed SFC− mice (45 days versus 31 days). The percentual change in sphingolipid ratios is indicated after the p value.

Short-term effects of SFC; comparison between SFC– and SFC+ mice three weeks after SFC

Brain region	SM16:0	SM18:0	SM20:0	SM22:0	SM24:0	SM24:1	Cer16:0	Cer18:0	Cer20:0	Cer22:0	Cer24:0	Cer24:1	Total SM/Total Cer	Total Cer/ Sphingosine
DH	T(16)=-0.015; p=0.988	T(15)=-0.100; p=0.922	T(16)=0.329; p=0.746	T(15)=-0.365; p=0.720	T(16)=-1.231; p=0.236	T(14)=-1.050; p=0.312	T(15)=-1.921; p=0.074	T(16)=1.618; p=0.125	T(15)=1.106; p=0.286	T(16)=-1.334; p=0.201	T(15)=-2.076; p=0.056	T(15)=-1.982; p=0.066	T(14)=0.381; p=0.709	T(15)=-1.524; p=0.148
HY	T(15)=0.095; p=0.925	T(16)=0.796; p=0.438	T(16)=1.370; p=0.190	T(15)=-1.303; p=0.212	T(16)=-0.484; p=0.635	T(16)=-0.913; p=0.375	T(15)=1.477; p=0.160	T(16)=0.816; p=0.427	T(15)=-0.492; p=0.630	T(15)=-1.109; p=0.285	T(16)=-0.519; p=0.611	T(15)=-1.245; p=0.232	T(14)=-1.173; p=0.260	T(15)=0.609; p=0.552
VH	T(16)=-1.432; p=0.171	T(15)=0.231; p=0.820	T(16)=-0.945; p=0.359	T(16)=-0.152; p=0.881	T(16)=-1.092; p=0.291	T(15)=-0.458; p=0.654	T(16)=-0.283; p=0.781	T(15)=1.064; p=0.304	T(16)=0.876; p=0.394	T(16)=-0.370; p=0.716	T(15)=-1.004; p=0.331	T(14)=-0.788; p=0.444	T(13)=0.658; p=0.522	T(14)=-0.299; p=0.769
VM	T(14)=1.450; p=0.169	T(15)=0.672; p=0.512	T(15)=-0.952; p=0.356	T(15)=0.397; p=0.710	T(14)=1.206; p=0.248	T(15)=-0.701; p=0.494	T(15)=0.975; p=0.345	T(14)=1.260; p=0.228	T(14)=-0.541; p=0.597	T(14)=-0.196; p=0.848	T(14)=0.376; p=0.713	T(14)=-2.131; p=0.051	T(14)=0.128; p=0.521	T(14)=1.299; p=0.215

Long-term effects of SFC; comparison between SFC– and SFC+ mice five weeks after SFC

Brain region	SM16:0	SM18:0	SM20:0	SM22:0	SM24:0	SM24:1	Cer16:0	Cer18:0	Cer20:0	Cer22:0	Cer24:0	Cer24:1	Total SM/Total Cer	Total Cer/ Sphingosine
FC	T(14)=-0.709; p=0.490	T(15)=-0.660; p=0.519	T(15)=0.440; p=0.666	T(14)=0.501; p=0.624	T(15)=-0.391; p=.0701	T(16)=0.089; p=0.930	T(15)=2.976; **p=0.009; -13%**	T(15)=-0.508; p=0.619	T(16)=-0.491; p=0.315	T(16)=0.009; p=0.993	T(16)=-0.219; p=0.829	T(16)=0.145; p=0.886	T(13)=-2.380; **p=0.033 ; +20%**	T(14)=3.774; **p=0.002; -26%**
AM	T(15)=-0.322; p=0.752	T(15)=1.202; p=0.248	T(14)=1.070; p=0.303	T(15)=-1.544; p=0.143	T(15)=0.229; p=0.822	T(14)=-0.658; p=0.521	T(15)=-0.111; p=0.913	T(15)=1.227; p=0.239	T(15)=1.362; p=0.193	T(15)=0.283; p=0.781	T(14)=-0.443; p=0.664	T(15)=-1.478; p=0.160	T(14)=-1.332; p=0.204	T(15)=1.130; p=0.276
DH	T(16)=0.686; p=0.502	T(15)=-1.069; p=0.302	T(15)=0.493; p=0.629	T(16)=0.778; p=0.448	T(16)=2.097; p=0.052	T(15)=1.077; p=0.299	T(16)=0.571; p=0.576	T(16)=-0.626; p=0.540	T(16)=0.293; p=0.773	T(15)=1.119; p=0.281	T(16)=0.593; p=0.561	T(15)=0.848; p=0.410	T(14)=-1.531; p=0.148	T(15)=-1.087; p=0.294
TH	T(14)=1.322; p=0.207	T(16)=2.908; **p=0.010; -10%**	T(15)=-1.341; p=0.200	T(16)=-1.360; p=0.193	T(16)=-1.017; p=0.324	T(16)=-3.328; **p=0.004; +43%**	T(16)=1.251; p=0.229	T(16)=4.363; **p<0.001; -3%**	T(16)=-1.673; p=0.114	T(15)=-1.189; p=0.253	T(15)=-1.244; p=0.233	T(14)=-3.865; **p=0.002; +38%**	T(12)=-1.839; p=0.091	T(13)=1.980; p=0.069
HY	T(16)=-1.554; p=0.140	T(15)=-1.072; p=0.301	T(16)=0.095; p=0.925	T(15)=0.357; p=0.726	T(15)=0.643; p=0.530	T(16)=1.255; p=0.227	T(16)=1.194; p=0.250	T(15)=0.856; p=0.406	T(16)=-1.853; p=0.082	T(16)=-1.611; p=0.127	T(16)=-1.525; p=0.147	T(16)=-0.311; p=0.760	T(15)=-2.459; **p=0.027; +25%**	T(15)=0.171; p=0.866
VH	T(16)=-0.482; p=0.636	T(15)=-0.790; p=0.442	T(15)=0.689; p=0.501	T(16)=-0.338; p=0.740	T(15)=-0.177; p=0.862	T(16)=1.162; p=0.262	T(16)=-0.560; p=0.583	T(16)=-0.323; p=0.751	T(16)=-0.074; p=0.942	T(16)=-0.478; p=0.639	T(15)=-1.899; p=0.077	T(15)=0.883; p=0.391	T(14)=0.619; p=0.815	T(15)=0.010; p=0.992
DM	T(16)=1.062; p=0.304	T(16)=-1.088; p=0.293	T(16)=1.656; p=0.117	T(14)=2.153; **p=0.049; -20%**	T(16)=1.693; p=0.110	T(14)=2.361; **p=0.033; -29%**	T(15)=-0.742; p=0.470	T(16)=0.629; p=0.538	T(16)=-1.670; p=0.114	T(16)=-1.039; p=0.314	T(15)=-1.283; p=0.219	T(16)=-0.020; p=0.985	T(13)=-0.396; p=0.699	T(14)=-0.745; p=0.469
VM	T(16)=1.646; p=0.119	T(15)=0.806; p=0.433	T(14)=-1.951; p=0.071	T(15)=-2.119; p=0.051	T(15)=-1.706; p=0.109	T(15)=-1.305; p=0.212	T(15)=1.163; p=0.263	T(15)=2.233; **p=0.041; -3%**	T(15)=2.519; **p=0.024; -16%**	T(16)=0.889; p=0.387	T(16)=-0.553; p=0.588	T(16)=-1.337; p=0.200	T(14)=-1.674; p=0.116	T(15)=0.994; p=0.336
CE	T(9)=-0.212; p=0.837	T(8)=-0.695; p=0.507	T(8)=-1.651; p=0.137	T(9)=0.447; p=0.666	T(9)=0.425; p=0.681	T(9)=1.056; p=0.319	T(9)=0.741; p=0.477	T(9)=-1.247; p=0.244	T(9)=-0.606; p=0.559	T(9)=-0.219; p=0.832	T(8)=-0.579; p=0.578	T(9)=1.140; p=0.284	T(8)=0.418; p=0.687	T(9)=0.285; p=0.783

Effects of duration of single housing; comparison between SFC– mice three weeks after SFC and SFC– mice five weeks after SFC

Brain region	SM16:0	SM18:0	SM20:0	SM22:0	SM24:0	SM24:1	Cer16:0	Cer18:0	Cer20:0	Cer22:0	Cer24:0	Cer24:1	Total SM/Total Cer	Total Cer/ Sphingosine
DH	T(16)=-1.418; p=0.175	T(16)=1.847; p=0.083	T(16)=-0.699; p=0.494	T(15)=-1.615; p=0.127	T(16)=-3.165; **p=0.006; +42%**	T(15)=-1.843; p=0.085	T(15)=-1.968; p=0.068	T(16)=2.609; **p=0.019; -2%**	T(16)=-1.629; p=0.123	T(16)=-3.488; **p=0.003; +22%**	T(16)=-2.520; **p=0.023; +15%**	T(15)=-2.265; **p=0.039; +28%**	T(15)=-2.049; p=0.058	T(15)=2.082; p=0.055
HY	T(15)=0.861; p=0.403	T(16)=-0.581; p=0.570	T(16)=0.232; p=0.820	T(14)=0.837; p=0.417	T(15)=0.684; p=0.504	T(16)=0.563; p=0.581	T(16)=-0.543; p=0.594	T(16)=-0.326; p=0.749	T(15)=-0.642; p=0.530	T(15)=-0.126; p=0.902	T(16)=0.489; p=0.632	T(15)=0.040; p=0.969	T(14)=0.065; p=0.949	T(15)=-0.448; p=0.660
VH	T(16)=-1.024; p=0.321	T(15)=1.465; p=0.163	T(16)=-0.595; p=0.560	T(16)=-0.137; p=0.893	T(16)=-0.656; p=0.521	T(16)=-1.556; p=0.139	T(16)=-0.252; p=0.805	T(15)=1.476; p=0.161.	T(16)=1.139; p=0.271	T(16)=0.286; p=0.778	T(15)=2.183; **p=0.045; -9%**	T(15)=-1.381; p=0.188	T(14)=0.488; p=0.633	T(15)=0.266; p=0.794
VM	T(16)=-0.292; p=0.774	T(15)=-1.512; p=0.151	T(15)=0.293; p=0.773	T(15)=2.882; **p=0.011; -32%**	T(15)=2.157; **p=0.048; -23%**	T(15)=2.724; **p=0.016; -31%**	T(16)=0.791; p=0.441	T(16)=-0.623; p=0.542	T(16)=-3.519; **p=0.003; +31%**	T(16)=-2.568; **p=0.021; +21%**	T(16)=-0.449; p=0.659	T(15)=1.330; p=0.203	T(15)=-0.990; p=0.338	T(16)=-0.412; p=0.686
